# Gender-based Barriers to the Advancement of Women in Academic Emergency Medicine: A Multi-institutional Qualitative Study

**DOI:** 10.5811/westjem.2021.7.52826

**Published:** 2021-10-26

**Authors:** Emily M. Graham, Meganne N. Ferrel, Katie M. Wells, Daniel J. Egan, Casey Z. MacVane, Michael A. Gisondi, Boyd D. Burns, Troy E. Madsen, Megan L. Fix

**Affiliations:** * University of Utah School of Medicine, Salt Lake City, Utah; † University of Vermont, Division of Emergency Medicine, Department of Surgery, Burlington, Vermont; ‡ Massachusetts General Hospital/Brigham and Women’s Hospital, Departments of Emergency Medicine, Boston, Massachusetts; § Maine Medical Center, Department of Emergency Medicine, Portland, Maine; ¶ Stanford University, Department of Emergency Medicine, Palo Alto, California; || University of Oklahoma School of Community Medicine, Department of Emergency Medicine, Tulsa, Oklahoma; # University of Utah, Division of Emergency Medicine, Department of Surgery, Salt Lake City, Utah

## Abstract

**Introduction:**

Leadership positions occupied by women within academic emergency medicine have remained stagnant despite increasing numbers of women with faculty appointments. We distributed a multi-institutional survey to women faculty and residents to evaluate categorical characteristics contributing to success and differences between the two groups.

**Methods:**

An institutional review board-approved electronic survey was distributed to women faculty and residents at eight institutions and were completed anonymously. We created survey questions to assess multiple categories: determination; resiliency; career support and obstacles; career aspiration; and gender discrimination. Most questions used a Likert five-point scale. Responses for each question and category were averaged and deemed significant if the average was greater than or equal to 4 in the affirmative, or less than or equal to 2 in the negative. We calculated proportions for binary questions.

**Results:**

The overall response rate was 55.23% (95/172). The faculty response rate was 54.1% (59/109) and residents’ response rate was 57.1% (36/63). Significant levels of resiliency were reported, with a mean score of 4.02. Childbearing and rearing were not significant barriers overall but were more commonly reported as barriers for faculty over residents (P <0.001). Obstacles reported included a lack of confidence during work-related negotiations and insufficient research experience. Notably, 68.4% (65/95) of respondents experienced gender discrimination and 9.5% (9/95) reported at least one encounter of sexual assault by a colleague or supervisor during their career.

**Conclusion:**

Targeted interventions to promote female leadership in academic emergency medicine include coaching on negotiation skills, improved resources and mentorship to support research, and enforcement of safe work environments. Female emergency physician resiliency is high and not a barrier to career advancement.

## INTRODUCTION

Gender disparities exist in academic emergency medicine (EM). Differences in compensation, slower career advancement, fewer tenured faculty positions, and discrimination are some of the challenges faced by women. These disparities have persisted for decades, despite increasing numbers of women entering the field and obtaining university appointments.^1^,^2^ Levels of career attrition are also higher when compared to men, which may also reflect a lack of career mentors, differences of support within and outside the workplace, gender bias, and discrimination.^3^–^6^ Heightened awareness of these disparities by individuals and institutions may facilitate solutions and ultimately improve patient care.^7^,^8^

As gender disparities are multifaceted, solutions from several vantages may be required to make an impact. Noteworthy interventions to reduce gender disparities in academic EM have been promoted in recent years. Professional society organizations are increasing awareness of gender disparities and developing leadership and career advancement resources for women. Additionally, numerous universities established resiliency centers, career mentoring programs, and policies to promote diversity, equity, and inclusion.^9^,^10^ Further defining the intrinsic factors contributing to gender disparities in medicine is also being explored by several specialties. Some of these factors include women physician wellness, resiliency, and risks of burnout.^11^–^13^ However, despite these efforts, significant gender disparity in academic EM persists. There also remains a gap in our understanding of the specific drivers of gender disparity in academic EM.

The objective of this multi-institutional survey study was to evaluate the degree of intrinsic motivators and extrinsic factors that impact the career trajectories of women in academic EM at the trainee and faculty level. By quantifying these factors, the experiences of women in academic EM can be better understood and may help identify areas needing continued improvement to better promote gender equality.

## METHODS

### Study Design and Population

This was a cross-sectional survey study of female-identifying faculty and residents in EM at eight academic medical centers in geographically distant regions of the United States. We performed sampling across the nation at multiple institutions to enhance generalizability and increase study power. Female-identifying participants were identified either by listserv or site investigator. A solicitation email described risks of study participation, and completion of the survey implied voluntary, informed consent. Anonymous responses were collected between November 2019–January 2020 using Google Forms (Alphabet Inc., Mountain View, CA) with reminders to non-respondents every two weeks until week six. The Institutional Review Board of the University of Utah approved the study.

### Survey Instrument and Methods

No previous investigation has examined all the domains we wished to explore; therefore, there was no validated instrument to use in this study. Accordingly, we developed an electronic survey tool based on expert opinion, literature review, and the lived experiences of women on our study team.^14^,^15^ Study investigators used iterative editing of the instrument to optimize internal structure evidence and content. Three investigators extensively tested the tool for item generation, optimal phrasing, matching of item content to the construct, survey functionality, and quality control. The survey was then piloted with medical students, residents, and faculty members at the University of Utah and was cross-checked for consistency to provide evidence of response-process validity. Final refinements of the instrument occurred in consultation with a PhD-level expert in survey-based research.

Participants were asked several demographic questions including race, ethnicity, geographic location of training program or current practice, and academic rank. We determined the primary outcomes of intrinsic motivators and extrinsic factors contributing to career advancement in two ways. First, participants were asked their agreement (1= strongly disagree; 5=strongly agree) with numerous statements that were categorized into five domains: self-determination; resiliency; career support and obstacles; career aspiration; and gender discrimination. Additional items that assessed gender discrimination, sexual assault, and/or battery in the workplace were asked as dichotomous yes/no questions. (Appendix 1, Survey Instrument.)

### Data Analysis

We analyzed data using Excel 2019 (Microsoft Corporation, Redmond, WA) and Origin 2018 (9.5 SR1) (OriginLab Corporation, Northampton, MA). Responses were analyzed by categorical dataset and as individual items. Means were calculated for each individual item and converted into a binary format with values of 1–3 signifying disagreement and responses with values of 4–5 signifying agreement. We reassigned demographic questions and other questions that required proportions into binary format for data analysis. Faculty responses were then compared to trainee responses using two-sided t-tests not assuming equal variance. We compared binary responses from faculty and residents using z-score calculations. Significance was determined with an alpha equal to or less than 0.05.

## RESULTS

Total response rate was 55.23% (95/172) with 59 faculty and 36 resident participants. The majority of respondents were non-Latinx Caucasians who trained in the northeast. Most faculty respondents held an assistant professor appointment. See Table 1 for a summary of respondent demographics. Figure A summarizes those items in which participants had significant agreement or disagreement. Most of these items were categorized in either the self-determination or resiliency domains, and these reflected participants’ strong commitment to their careers and achievement of their goals. Most participants agreed that they had enough family support to advance their careers, while only half of participants were aware of career mentoring programs at their institutions. Importantly, 68.4% of respondents experienced gender discrimination and 9.5% experienced sexual assault and/or battery by colleagues or supervisors (Figure B). Notably, 58.0% of participants had never been the primary investigator (PI) of a project, 75% of participants had never written a grant, and only 18% of participants reported feeling comfortable with work-related negotiations.

There were significant differences between faculty and resident respondents. Faculty members were less likely to change jobs to advance their careers, with response average of 3 for faculty and 3.67 for residents (*P*<0.01), had fewer career mentors with a faculty average of 3.14, residents 3.75 (*P* = 0.03), and were more comfortable negotiating with superiors for salary and paid time off, faculty response 2.54, residents 2.02 (*P* = 0.03). Additionally, faculty respondents more commonly identified childbearing/child rearing as a reason for a stunted career, with a faculty response of 2.78 and resident response of 1.69 (*P* <0.001), and more commonly sacrificed career advancement for family or personal reasons, with a faculty response of 3.0, resident response of 2.14 (*P* = 0.001). Of note, 38.8% of participants did not hold any leadership positions.

## DISCUSSION

This study provides additional insights about the causes of career disparities experienced by women in academic EM, specifically identifying the need for improved training in employment negotiation and research productivity. Our findings are consistent with previously published reports^1^,^7^ of factors most strongly tied to disproportionate professional attrition and lack of equal representation. Our respondents did not identify lack of career support as a barrier to advancement, unlike other published studies. While many explanations may explain this finding, a reasonable explanation includes increased support from family or others to improve quality of life outside of work. Finally, we confirmed the previously reported need for gender equitable policies at the institutional level.^1^,^4^

Importantly, our study participants reported high levels of resiliency. Similarly, we did not identify resiliency as a meaningful barrier to career advancement. Becoming an emergency physician takes resiliency, and choosing to remain on the frontlines of medicine shows ample dedication and perseverance. However, since physician burnout remains prevalent, many institutions continue concluding that wellness initiatives are the major solution. In addition to current reports, our findings support that while resiliency centers and physician wellness programs are meaningful, they are not the only solution. Improving system issues requires equal attention and effort. Thus, interventions to improve career advancement should assume a resilient workforce and instead focus on causes external to the individual. Strategies to improve the work milieu include decreasing administrative burdens, increasing physician autonomy, ensuring safe work environments, and providing resources for extra-clinical duties.

Advancement to leadership positions may be largely influenced by research productivity throughout an academic career.^4^ Our findings confirm the importance of successful scholarship and identifies the need to better support women in EM to conduct research, as many respondents reported inexperience as a PI and with grant writing. Interventions that prioritize research mentorship and training for women faculty are warranted.

A disturbing, unexpected study finding was the reported incidence of gender discrimination and sexual assault in our cohort of women emergency physicians. A majority of participants experienced gender discrimination from their colleagues and/or supervisors at some point in their careers, with 1 in 10 respondents also suffering sexual assault and/or battery. These rates exceed those in a 2018 seminal report by the National Academies estimating that 50% of women physicians experienced sexual harassment at work, an incidence second only to women in the military.16 Further exploration with large cohorts is required to determine whether our findings highlight a longstanding, unspoken reality specific to the specialty of EM.

Differences in perceived barriers to career advancement between faculty and resident physicians were notable, and our findings suggest that certain barriers may have improved over time. For example, faculty members were less likely to have a female mentor as compared to residents. This may be a simple function of the availability of female mentors at different career stages, with a lack of senior faculty members available to mentor junior faculty. In addition, faculty more frequently reported that childbearing/parenting negatively impacted their career than residents. The same held true regarding the sacrifice of family or personal life for career. Finally, residents were more optimistic about their ability to achieve a successful work-life integration in the face of new leadership opportunities.

Looking forward, based on our study findings we propose the following areas of focus for departments and institutions to improve gender equity in academic EM: 1) establish gender equitable policies on an institutional level; 2) decrease administrative burdens; 3) increase physician autonomy; 4) ensure safe work environments; 5) provide resources for extra-clinical duties (ie, research). These also represent areas ripe for future research.

## LIMITATIONS

Despite a multi-institutional study design, the limited number of women physicians available to participate in the study impacts the generalizability of our findings and may introduce bias. We addressed this issue somewhat by sampling respondents from all regions of the country. However, despite our efforts to poll a diverse group of women physicians in EM, the majority of respondents identified as Caucasian. Future studies are needed to elucidate how race and gender impact career advancement.

Future studies may also choose to explore how academic rank impacts responses, since the majority of our respondents were of the assistant professor rank. Participants who completed the survey likely also had an interest in the topic, which may have furthered sampling bias and impacted results. Although the rates of gender discrimination and sexual assault were higher than anticipated, another limitation to this study may include reporting bias as many are uncomfortable disclosing these encounters in a survey. Additionally, there was no validated survey tool available to use for our survey. Hence, as with any new survey instrument there is also a lack of established validity and reliability of our tool for our study cohort. Finally, this study was limited by the inclusion of only female participants, which did not allow for a male comparison group.

## CONCLUSION

Our study found that previously identified barriers to career advancement by women in academic EM, such as poor resiliency or the demands of parenting, may not be as significant as in the past. Instead, obstacles related to employment negotiations and research experience are more contemporary issues requiring gender specific interventions. Our study also revealed unexpectedly high incidences of gender discrimination and sexual assault that are unacceptable and mandate an immediate, large, cohort-replication study.

## Supplementary Information



## Figures and Tables

**Figure:**
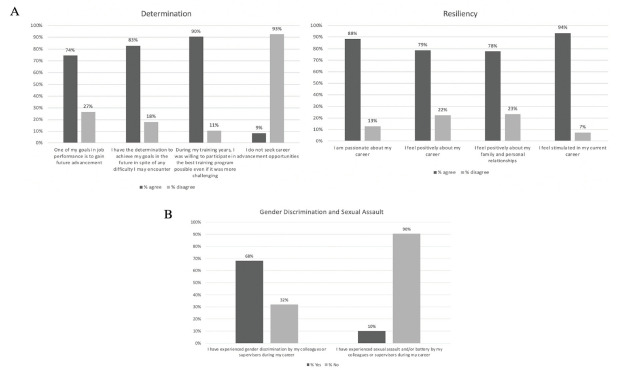
Combined findings of residents and faculty members 1A: Categorical groupings of residents and faculty members in determination and resiliency results; 1B: Those who experienced gender discrimination and sexual assault.

**Table:** A summary of the demographic information from women faculty and residents in emergency medicine.

	n	(%)
Race (n=95)		
White/Caucasian	75	78.9
Latinx	3	3.2
Asian	11	11.6
African American	3	3.2
Native Alaskan/Native American	1	1.1
Other/Unspecified	2	2.1
Faculty academic rank (n=59)		
Assistant Professor	47	79.7
Associate Professor	6	10.2
Full Professor	3	5.1
None	3	5.1
Highest leadership position held by faculty (n=59)		
Committee Leader	6	10.2
Medical Director	3	5.1
Program Director	4	6.8
Division Chief	2	3.4
Department Chair	0	0
None	44	74.6
Location of training (n=95)		
Midwest	18	18.9
Northeast	34	35.8
Southeast	18	18.9
Southwest	5	5.3
West	18	18.9
Outside of the United States	2	2.1
