# Supplementary material for: Gender-based Barriers to the Advancement of Women in Academic Emergency Medicine: A Multi-institutional Qualitative Study
Source: West J Emerg Med. 2021 Oct 26;22(6):1355–9. doi: 10.5811/westjem.2021.7.52826 (PMC8597699; doi:10.5811/westjem.2021.7.52826)
Supplement: Supplementary file 1 [file wjem-22-1355-s001.docx]

**Appendix**. Survey instrument.

1. Where did you do medical school?
2. Which of the following describes the number of years you have been in practice since your graduation from medical school?
   1. Current Resident
   2. < 5 years
   3. 5-9 years
   4. 10-14 years
   5. 15-19 years
   6. 20-24 years
   7. 25-29 years
   8. 30-34 years
   9. 35-40 years
   10. >40 years
3. Which of the following best describes your highest position held in your career? (more than one option accepted)
   1. Assistant professor
   2. Associate professor
   3. Full professor
   4. Committee Leader
   5. Medical Director
   6. Program Director
   7. Department Chair
   8. Division Chief
   9. None of the above
4. What is your specialty? (If you are still in your training, type 'NA')
5. Please select the options that best describe your racial and ethnic background:
   1. White/ Caucasian
   2. Latinx
   3. Asian
   4. Black or African American
   5. Native Alaskan or Native American
   6. Pacific Islander
6. If you have a career mentor, how did you find them?
7. Does your institution offer a formal career mentoring program?
   1. Yes
   2. No
8. Likert 5-point scale questions: 1 being strongly disagree and 5 being strongly agree
   1. Determination
      1. One of my goals in job performance is to gain future advancement
      2. I am confident in my ability to apply for leadership positions within my institution
      3. I have the determination to achieve my goals in the future in spite of any difficulty I may encounter
      4. I am willing to transfer to a better workplace to advance my career
      5. During my training years, I was willing to participate in the best training program possible even if it was more challenging
      6. I do not seek career advancement opportunities
   2. Resiliency
      1. I am passionate about my career
      2. I have achieved a work-life balance
      3. I feel positively about my career
      4. I feel positively about my family and personal relationships
      5. I would choose a career in medicine again
      6. I feel stimulated in my current career
   3. Support
      1. I have a female mentor who provides career advice and sponsorship
      2. I have a male mentor who provides career advice and sponsorship
      3. I have a female confidant with whom I easily consult in my institution
      4. I have a male confidant with whom I easily consult in my institution
      5. My supervisors and colleagues are understanding and supportive of my situation and motivation for my career
      6. I have enough family support to advance my career
   4. Career Aspiration
      1. I actively search and apply for leadership positions within my field of medicine
      2. To enhance my career, I am willing to participate in various meetings and interact with others
      3. I am/have been the PI of at least one major research project
      4. I have received a grant for research as a PI
      5. I am comfortable negotiating with my supervisors (salary, paid time off, etc.)
   5. Obstacles
      1. My career has been significantly stunted due to childbirth and child rearing
      2. I sacrificed career advancement for my family or personal life
      3. I am not provided opportunities to prove myself in my career
      4. Few female role models in leadership contributes to the low numbers of females in academic medicine
      5. It would be impossible to maintain my work-life balance if I become a leader now
   6. Gender Discrimination
      1. I am capable of addressing gender discrimination in my Department/Division/Training Institution?
      2. Women have difficulties advancing in their careers compared to men, regardless of merit or talent
      3. Male physicians are less likely to take orders from female superiors
      4. Supporting staff (nurses, techs, pharmacists) are less likely to take orders from female physician superiors
      5. Being female is an advantage in medicine because women stand out
9. I have experienced gender discrimination by my colleagues or supervisors during my career
   1. Yes
   2. No
10. I have experienced sexual assault and/or battery by my colleagues or supervisors during my career
    1. Yes
    2. No
11. What helpful practices should medical students who are women or a minority gender do to develop resiliency?
12. What advice would you provide to medical students who are women or a minority gender pursuing an academic career in medicine?
13. What are essential skills a leader in medicine should develop in order to lead effectively?
14. What resources would you advise medical students who are women or a minority gender use to better prepare for leadership positions?
15. Please add any additional thoughts or comments.
